# N-terminal formylmethionine as a degron and a specific signal in proteostasis and stress adaptation

**DOI:** 10.1038/s12276-026-01723-1

**Published:** 2026-05-08

**Authors:** Chang-Seok Lee, Dasom Kim, Cheol-Sang Hwang

**Affiliations:** 1 Department of Life Sciences, Korea University, Seoul, Republic of Korea; 2 National Research Laboratory for Convergence Degradation Biology, Korea University, Seoul, Republic of Korea

**Keywords:** Protein quality control, Protein quality control, Ubiquitylation

## Abstract

N-terminal (Nt) methionine formylation, once thought restricted to bacteria and organelles, is now recognized as a stress-inducible initiator modification in the eukaryotic cytosol. Under metabolic or environmental stress, mitochondrial methionyl-transfer RNA (tRNA) formyltransferase mislocalizes to the cytosol, generating formylated initiator tRNA (fMet-tRNAi) that initiates translation with *N*-formylmethionine (fMet). Nascent chains bearing Nt-fMet activate an fMet-directed ribosome-associated quality control checkpoint early in elongation, recruiting ribosome-splitting and disaggregation factors. Stalled complexes are routed to stress granules, conserving mRNA, translation machinery, and energy, while limiting aggregation. During prolonged stress, newly synthesized fMet proteins undergo maturation or selective degradation via the fMet/N-degron pathway. In mammals, E3 ligase TRIM52 acts as an Nt-fMet recognin, modulating apoptosis. Proteolytic clearance of cytosolic fMet substrates releases formylated peptides and free fMet, which are elevated in critical illness and activate formyl peptide receptors — linking translation surveillance to innate immune and inflammatory signaling in sepsis and age-related disease. Advances in N-terminomics and anti-fMet reagents now allow direct detection and quantification of cytosolic fMet proteoforms. This Review integrates bacterial and organellar paradigms with emerging cytosolic mechanisms, examines regulatory gating of Nt-formylation, and highlights therapeutic strategies to restore proteostasis and counter fMet-associated pathology.

## Proteostasis: N-terminal formylmethionine supports stress adaptation

N-terminal methionine formylation, a protein modification once thought to be exclusive to bacteria and organelles, also occurs on proteins synthesized in the cytosol of yeast and human cells, challenging long-held assumptions. This Review describes how formylmethione (fMet) contributes to protein synthesis, stability, and cellular stress responses, particularly through the fMet-ribosome quality control and fMet/N-degron pathways. By coupling the stress-induced synthesis of cytosolic fMet-containing proteins to their selective removal, these pathways help maintain proteostasis and promote cellular adaptation. The findings highlight the evolutionary conservation of N-terminal methionine formylation and its potential implications in stress adaptation, cancer, and immune signaling. Future studies should identify Nt-formylated proteins across different conditions and explore therapeutic potential of targeting this modification.

This summary was initially drafted using artificial intelligence, then revised and fact-checked by the author.

## Introduction

Nα-terminal (Nt) modifications are key determinants of protein fate, influencing folding, activity, localization, and stability across all domains of life^1–3^. Among these, Nt-formylmethionine (Nt-fMet) is a hallmark of bacterial and organellar translation, in which it initiates protein synthesis and orchestrates early co-translational processing^4–7^. Beyond initiation, Nt-fMet contributes to translational fidelity, co-translational folding, protein stability, the assembly of multiprotein complexes, and membrane integrity^8–11^.

For decades, Nt-fMet-directed protein synthesis in eukaryotic cytosol was considered absent or negligible, largely due to the presumed lack of a cytosolic methionyl-transfer RNA (tRNA) formyltransferase (FMT) and technical barriers to detection^5,12^. However, recent advances in Nt-proteomics^13^ and the development of pan-fMet antibodies have revealed robust synthesis of fMet-bearing proteins on cytosolic ribosomes in both yeast and human cells^14–17^. These discoveries uncovered a ribosome quality control (RQC) mechanism directed by Nt-fMet (the fMet-RQC pathway)^18^ and a proteolytic degradation route targeting Nt-fMet (the fMet/N-degron pathway)^14,17^.

In parallel, stress-induced cytosolic fMet-protein synthesis, along with the fMet-RQC and fMet/N-degron pathways, implies an endogenous pool of N-formylated peptides (formyl peptides) within eukaryotic cells^19^. These peptides, previously attributed solely to bacterial or mitochondrial origins^20–22^, may activate formyl peptide receptors (FPRs), thereby modulating innate stress responses and cellular signaling^19^.

This Review highlights recent advances in cytosolic fMet-protein synthesis and its regulation through the fMet-RQC and fMet/N-degron pathways. Topics specific to bacterial and organellar translation, FPR signaling, and therapeutic implications are discussed in detail elsewhere^6,7,21–23^.

## Protein Nt-formylation

### Mechanistic diversity of protein formylation

**Fig. 1:**
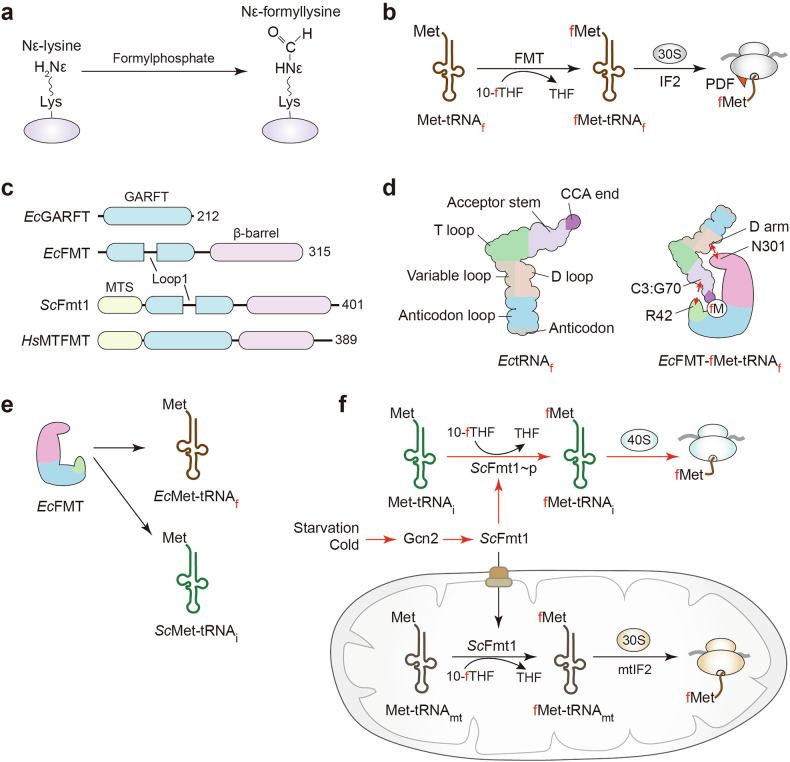
Eukaryotic cytosolic fMet-protein synthesis and regulatory features of formyltransferase. **a** Chemical conversion of ε-lysine to Nε-formyllysine by formylphosphate derived from DNA or formaldehyde. To date, no dedicated Nε-deformylase has been reported in vivo. **b** Canonical pathway of Nt-Met formylation. Met-tRNA_f_ is converted to fMet-tRNA_f_ by formyltransferase (FMT) using 10-fTHF as a one-carbon donor. IF2 then delivers fMet-tRNA_f_ to the small subunit ribosome. Following translation initiation, PDF deformylase removes the formyl group. **c** Domain organizations of bacterial (*Escherichia coli*), yeast (*Saccharomyces* *cerevisiae*), and human (*Homo sapiens*) FMTs and the related GARF homolog. *Sc*Fmt1 and *Hs*MTFMT contain a mitochondrial targeting sequence (MTS). **d** Recognition of initiator Met-tRNA_f_ by *Ec*FMT. Left: *Ec*tRNA_f_ with key determinants for formylation. Right: the *Ec*FMT catalytic pocket highlighting contacts between R42 and the tRNA C3:G70 base pair, and between N301 and the tRNA D arm. **e** Substrate specificity of *Ec*FMT. *Ec*FMT efficiently recognizes its cognate *E. coli* Met-tRNA_f_ and, to a lesser but detectable extent, the eukaryotic *S. cerevisiae* Met-tRNAi. **f** Dual localization and stress-dependent activation of *Sc*Fmt1. *Sc*Fmt1 is constitutively mitochondrial, generating fMet-tRNA_mt_ for mitochondrial translation. Upon nutrient depletion or cold stress, Gcn2-dependent phosphorylation retains *Sc*Fmt1 in the cytosol, where it utilizes 10-fTHF to produce cytosolic fMet-tRNAi for non-canonical initiation in the cytosol. GARFT, glycinamide ribonucleotide formyltransferase; MTFMT, mitochondrial FMT; PDF, peptide deformylase; THF, tetrahydrofolate; tRNA, transfer RNA.

Protein formylation arises via two distinct processes. Nε-lysine formylation is a non-enzymatic, damage-associated adduct, in which reactive metabolites (for example, 3′-formylphosphate from oxidative DNA damage or formaldehyde) modify lysine side chains^24,25^ (Fig. 1a). Low-level Nε-formyllysine is detected on histones and can compete with acetylation, but its in vivo roles remain unresolved^25^.

By contrast, Nt-fMet is generated pre-translationally when FMT transfers a formyl group from 10-formyltetrahydrofolate (10-fTHF) onto the α-amine of initiator Met-tRNA, yielding fMet-tRNA for initiation^6,7^ (Fig. 1b). Alternative one-carbon donors such as 10-formyldihydrofolate also support FMT activity, linking formylation to folate metabolism and antifolate sensitivity^26^. For clarity, initiator tRNAs are, hereafter, denoted tRNA_f_ (bacteria), tRNA_mt_ (mitochondria), and tRNA_i_ (cytosol).

### Catalytic mechanism and structural features of FMT

FMT transiently formylates the Nt-α-amine, thereby licensing downstream processing events: peptide deformylase (PDF) removes the formyl group before sequence-directed Nt-Met excision and/or subsequent modification such as Nt-acetylation^1,2^. FMT shares functional hallmarks with other folate-dependent FMTs, including glycinamide ribonucleotide formyltransferase, such as substrate-induced conformational changes and formyl transfer via an activated carbon intermediate^27^ (Fig. 1c).

Bacterial FMTs comprise an N-terminal Rossmann-like catalytic domain and a C-terminal β-barrel tRNA-binding domain^27^. In *Escherichia coli*, a 16-residue insertion loop docks onto the initiator tRNA acceptor stem at the hallmark C1·A72 mismatch, positioning the methionylated 3′ end into the catalytic cleft through an induced-fit mechanism^27,28^ (Fig. 1d). Efficient substrate recognition relies on conserved tRNA identity elements (G2–C71, C3–G70, G4–C69, and A73) and a conserved arginine in the insertion loop (for example, *E. coli* FMT Arg42)^29,30^ (Fig. 1d).

These structural features are conserved in both *E. coli* Met-tRNA_f_ and *Saccharomyces cerevisiae* initiator Met-tRNAi, enabling cross-species recognition. Consistently, ectopic expression of *E. coli* FMT in yeast results in efficient formylation of the endogenous Met-tRNAi and implies fMet-initiated translation in the cytosol of yeast cells^31^ (Fig. 1e).

### FMT substrate compatibility and compartmental specificity

Despite conserved overall folds, FMT substrate preferences differ by lineage and compartment. In budding yeast, the mitochondrial *Sc*Fmt1 stringently recognizes initiator-type identity in the acceptor stem (notably the non-Watson–Crick 1–72 signature) and, accordingly, does not act on the cytosolic Met-tRNAi, which carries eukaryotic initiator features instead of the bacterial-type C1•A72 mismatch^32^.

Under stress (for example, cold shock or nutrient starvation), a fraction of *Sc*Fmt1 relocalizes to the cytosol, enabling conditional Nt-formylation outside the organelle^14,18^. In metazoan mitochondria, in which a single Met-tRNA_mt_ services both initiation and elongation, MTFMT relies more on the methionyl moiety and limited stem cues than on bacterial-type identity elements^33^. Pathogenic MTFMT variants impair Nt-formylation and mitochondrial translation, causing Leigh syndrome with oxidative phosphorylation (OXPHOS) defects^22,34,35^. Thus, initiator tRNA sequence, subcellular localization, and lineage-specific FMT recognition shape the spatial and temporal dynamics of Nt-formylation across eukaryotes and their organelles^6,22,36^.

### FMT conservation and functional divergence

Formylation of Met-tRNA_f_ enhances bacterial initiation by favoring high-affinity binding to initiation factor IF2, excluding elongation factor EF-Tu, and stabilizing P-site loading on the ribosome^6,7,37^ (Fig. 1b). In *E. coli*, loss of FMT slows growth and compromises initiation fidelity, whereas loss or inactivation of PDF is lethal or severely deleterious, underscoring the essential nature of the formylation–deformylation cycle^7,11,38^.

A parallel mechanism operates in mitochondria: the mitochondrial IF2 (mtIF2) preferentially binds fMet-tRNA_mt_^39^. Although translation can proceed to some extent with non-formylated Met-tRNA_mt_, deficiency of MTFMT reduces translational efficiency, destabilizes OXPHOS complexes I/IV, and impairs mitochondrial proteostasis^22,35,40^. By contrast, archaea lack FMT and initiate with unmodified Met-tRNAi delivered by a/eIF2; an IF2 ortholog (aIF5B) acts later to promote subunit joining — an evolutionary bypass of Nt-formylation^41,42^.

Similarly, obligate endosymbionts with extreme genome reductions, such as mealybug-associated symbionts, have independently lost both FMT and PDF. These lineages compensate by importing host or co-symbiont factors or by relaxing their dependence on Nt-formylation altogether, illustrating a flexible reconfiguration of the translation initiation machinery^43–45^.

### Cytosolic Nt-formylation in eukaryotes

In eukaryotes, FMTs are nuclear-encoded and primarily targeted to mitochondria (and plastids in plants), reflecting their bacterial ancestry. Nevertheless, under specific stresses (for example, nutrient limitation and cold) and in some cancer contexts (for example, colorectal cancer), FMT can accumulate in the cytosol, enabling fMet-initiated translation outside organelles^14,15,18,46^.

Consistent with possible extra-mitochondrial pools, the Human Protein Atlas annotates human mitochondrial FMT (MTFMT) as mainly nucleoplasmic with additional cytosolic localization across cell lines^47^. Subcellular fractionation in certain human cells detected fMet-bearing proteins and MTFMT in both mitochondrial and cytosolic fractions^48^. However, the cellular cues, which might drive any extra-mitochondrial relocalization of MTFMT, and the physiological role of such pools remain to be determined.

In *S. cerevisiae*, starvation or entry into stationary phase triggers Gcn2 (eIF2α kinase)-dependent phosphorylation that retains a fraction of *Sc*Fmt1 in the cytosol, increasing cytosolic Nt-formylation and enabling stress-responsive initiation^14^. In parallel, Gcn2-mediated phosphorylation of eIF2α dampens bulk translation^49^ while permitting selective fMet-initiated programs decoupled from global protein synthesis^14^ (Fig. 1f).

In mammals, GCN2 (EIF2AK4) is activated by uncharged tRNAs and ribosome collisions; GCN1 recruits GCN2 to collided disomes, and contacts with the ribosomal P-stalk relay the collision signal to the integrated stress response (ISR)^49,50^. When eIF2 is inhibited by eIF2α phosphorylation, alternative initiation routes engage: eIF2D and the MCTS1·DENR heterodimer promote re-initiation/initiator-tRNA delivery on upstream open reading frame (uORF)-programmed transcripts (including ATF4), and eIF5B can support eIF2-independent initiation, especially under stress or internal ribosome entry site/non-AUG contexts; by contrast, eIF2A contributes little to global or uORF-mediated initiation in human cells^51–53^. These conserved GCN2 signaling and eIF2-bypass routes may regulate cytosolic retention of MTFMT and fMet-initiated translation^48^. It also remains plausible that other regulatory pathways modulate fMet levels and MTFMT localization or activity.

## Protein deformylation

### PDF catalysis and specificity

PDF is a ribosome-associated mononuclear metallohydrolase that removes the Nt-formyl group co-translationally as nascent chains emerge the exit tunnel^2^ (Fig. 1b). Structural and dynamics studies place PDF on the large ribosomal subunit near uL22, consistent with in situ, on-ribosome catalysis^54,55^. The active site of PDF is built around three conserved motifs — GXGXAAXQ, EGCLS, and HEXXH — that chelate the catalytic metal and position the nucleophilic water; Fe^2+^ is the physiological cofactor (activity can be retained with Ni^2+^ in vitro)^56^. These motifs underpin high selectivity for the small formyl group while excluding bulkier acyl substituents. Substrate recognition relies largely by backbone interactions at the N terminus, which explains broad tolerance of PDFs for diverse +1/+2 residues across bacterial proteomes^57^.

On translating ribosomes, PDF competes kinetically with signal recognition particle (SRP): SRP binding to signal-sequence nascent chains shields the fMet and delays deformylation, introducing a timing checkpoint before membrane targeting^58^. When deformylation is delayed or blocked, formylated N termini accumulate and trigger stress and quality control responses in bacteria. Acute PDF inhibition rapidly induces proteostasis and membrane stress programs^11^.

### PDF conservation, functional relevance, and therapeutic implications

PDF is a ubiquitous and essential enzyme in bacteria, where it co-translationally removes the Nt-formyl group from nascent polypeptide chains, a critical step for proper protein maturation and function^2,11^. Homologous enzymes are retained in many eukaryotic organelles of bacterial origin, namely, mitochondria and plastids, where translation initiates with fMet. By contrast, the eukaryotic cytosol and archaea do not utilize fMet and generally lack canonical PDF^41,59–61^.

Notably, canonical PDF genes are absent from the genomes of *S. cerevisiae* and *Caenorhabditis elegans*, indicating lineage-specific losses of deformylation capacity in some eukaryotes^60^. Eukaryotes with reduced or absent mitochondria also lack PDF: *Giardia* possesses mitosomes without organellar translation, and *Monocercomonoides* has secondarily lost mitochondria altogether^62^. Remarkably, giant marine viruses encode their own PDFs within specialized translational modules, co-opting the formylation–deformylation cycle to ensure proper processing of viral proteins^63,64^.

In mammals, mitochondrial PDF (mtPDF) facilitates efficient synthesis and assembly of OXPHOS complexes. Genetic or pharmacological inhibition of mtPDF reduces the expression of mtDNA-encoded subunits and impairs respiratory function, highlighting the organelle-restricted yet essential role of deformylation in mitochondrial bioenergetics^65^. Furthermore, mtPDF is upregulated in several tumor types, suggesting its relevance in cancer metabolism^66^.

The strict requirement for PDF in bacteria, combined with its absence from the eukaryotic cytosol, has made it an attractive antibacterial target^67,68^. Actinonin-class inhibitors that block the PDF active site lead to toxic accumulation of formylated proteins and collapse bacterial proteostasis^11,69,70^. Although early compounds suffered from poor bioavailability and off-target effects, next-generation PDF inhibitors with enhanced specificity, pharmacokinetics, and reduced toxicity are now in development. Selective inhibition of mtPDF may also hold as an anticancer strategy, particularly in tumors reliant on organellar translation and OXPHOS activity^68,71^.

## The fMet-RQC pathway: a novel ribosome surveillance system

### Canonical RQC pathways

**Fig. 2:**
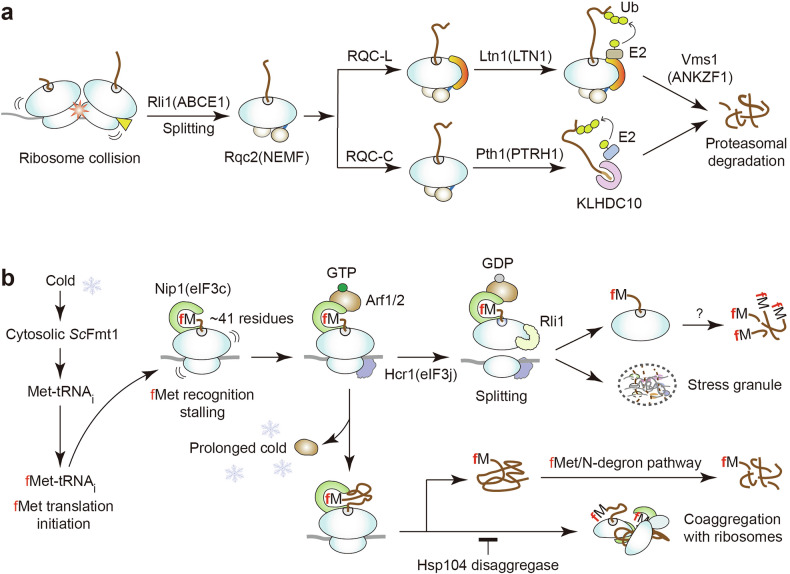
Canonical RQC and fMet-RQC pathways. **a** Canonical RQC in yeasts and mammals. Collisions from stalled ribosomes or damaged mRNAs (rare codons, polybasic tracts, premature stops, drug-induced pausing, ribosome defects) trigger subunit splitting by Rli1 (yeast) or ABCE1 (mammals), with Rqc2 (NEMF) stabilizing the 60S–peptidyl-tRNA complex. Resolution proceeds via two branches. RQC-L: Ltn1 (LTN1) ubiquitinates the nascent chain; release is catalyzed by Vms1 (ANKZF1), followed by proteasomal degradation. RQC-C: peptidyl-tRNA hydrolysis by Pth1 (PTRH1) couples to C-degron surveillance; in mammals, the CRL2 receptor KLHDC10 recognizes Ala-tail/C-degron substrates, with no established budding-yeast counterpart^79,80,151^. **b** The yeast fMet-RQC pathway. Cold stress retains *Sc*Fmt1 in the cytosol, increasing the production of fMet-tRNAi from Met-tRNAi. When approximately 41 residues of an fMet-initiated nascent chain emerge from the exit tunnel, its Nt-fMet is sensed by Nip1 (eIF3c), which recruits Arf1/Arf2 GTPases to induce ribosome stalling. Stalled ribosomes then recruit Hcr1 (eIF3j) and the recycling factor Rli1, which splits subunits. The resulting 60S–peptidyl-tRNA species bearing fMet chains are extracted by as-yet unknown factors and degraded by the proteasome (mechanism unresolved), whereas 40S–mRNA species partition into stress granules to conserve metabolic resources and preserve mRNAs and translation components. During prolonged cold, Arf1/Arf2 dissociates from stalled ribosomes, allowing continued elongation and accumulation of fMet-initiated proteins that are subsequently cleared by the fMet/N-degron pathway. Under these conditions, Nt-fMet-bound Nip1 can co-aggregate with ribosomal subunits; such aggregates are only partially resolved by the Hsp104 disaggregase^18^. RQC, ribosome quality control; tRNA, transfer RNA.

Aberrant ribosome stalling, caused by defective mRNAs, rare codons, polybasic tracts, or drug-induced pauses^72–76^, generates incomplete nascent chains that contribute to cellular stress, aging, and neurodegeneration^77–82^. Canonical RQC pathways mitigate these stressors by detecting stalled ribosomes, splitting subunits, and routing aberrant chains for degradation^74,79–81^ (Fig. 2a).

Ribosome stalling causes ribosome collisions, which activate surveillance pathways such as no-go decay, non-stop decay, and non-functional rRNA decay^72,75,80,83^ (Fig. 2a). Collided ribosomes are selectively ubiquitinated on small subunit proteins by the E3 ligase Hel2 (ZNF598), which is an essential step for the canonical RQC activation^84,85^. This Hel2 (ZNF598)-dependent ubiquitination initiates ribosome rescue through two distinct routes: (i) the Dom34 (PELO)–Hbs1 (HBS1L)–Rli1 (ABCE1) pathway, in which Dom34/PELO mimics peptidyl-tRNA-hydrolysis-lacking eRF1 and probes stalled ribosome, Hbs1/HBS1L provides GTPase-driven ribosome conformational transition, and Rli1/ABCE1 (an ATPase) powers subunit splitting and recycling^86,87^, and (ii) the RQT (RQC trigger) pathway, composed of the RNA helicase/ATPase Slh1 (ASCC3), the ubiquitin-binding adaptor Cue3 (ASCC2), and the zinc-finger scaffold Rqt4 (TRIP4), which recognizes ubiquitinated stalled ribosomes and drives their dissociation^88,89^. (Mammalian counterparts of yeast RQC components are indicated in parentheses.)

Following subunit splitting by Rli1 (ABCE1), assisted by ATP hydrolysis and the tRNA/nascent-chain-binding factor Rqc2 (NEMF), 60S–peptidyl-tRNA complexes are processed through two branches. In the RQC-L (large subunit) branch, Rqc2 catalyzes C-terminal alanine/threonine (CAT) tailing^90^, Ltn1 (LTN1/Listerin) ubiquitinates the stalled chain^73^, and the peptidyl-tRNA hydrolase Vms1 (ANKZF1) hydrolyzes the peptidyl-tRNA bond, enabling proteasomal degradation^76,91^. In the RQC-C branch, peptidyl-tRNA hydrolase Pth1 (PTRH1) and C-end rule E3s — particularly CRL2–KLHDC10 and PIRH2 — target nascent peptides for degradation through recognizing C-terminal degrons (including Ala-tails)^92–94^ (Fig. 2a).

### Cytosolic Nt-formylation triggers a new RQC mechanism

In yeast, cytosolic translation normally initiates with unmodified Met. However, under stress conditions such as cold exposure or nutrient deprivation, and upon ectopic expression of *E. coli* FMT, Met-tRNAi can be enzymatically converted into formylated fMet-tRNAi, initiating translation with fMet^14,18^. Because yeast lacks a PDF^60^, fMet-initiated proteins can accumulate and become potentially toxic. Notably, overexpression of *E. coli* FMT in *S. cerevisiae* converts up to ~70% of Met-tRNAi to fMet-tRNAi^31^. Despite this high conversion rate, Nt-formylated proteins represent only ~7% of detected protein species, and only ~5% of these are degraded by the fMet/N-degron pathway^14^. This discrepancy points to an additional co-translational quality control mechanism that limits the toxic buildup of fMet-bearing nascent chains^18^. Both ectopic *E. coli* FMT expression and cold-induced cytosolic retention of *Sc*Fmt1 similarly produce fMet–tRNA_i_, triggering fMet-dependent translation on cytosolic ribosomes. This activates the fMet-RQC pathway, a specialized branch of RQC distinct from canonical mechanisms^18^.

As the Nt-formylated nascent chain exits the ribosomal tunnel (around codon 41), elongation stalls. This stall is initiated by the recognition of nascent Nt-fMet by Nip1 (eIF3c), a translation initiation factor that localizes from the 40S to the 60S subunit, where it directly binds Nt-fMet. This event triggers a surveillance cascade that is independent of ribosome collision or mRNA damage^18^ (Fig. 2b). Nip1 recruits Arf1/Arf2, small GTPases of the ARF/ARL family, together with the recycling factor Rli1, likely via Hcr1 (eIF3j, a subunit of eukaryotic translation initiation factor eIF3). The GTPase activity of Arf1/Arf2 promotes Rli1-mediated subunit dissociation and also recruits the disaggregase Hsp104, thus preventing co-aggregation of short fMet-bearing nascent chains with ribosomes^18^ (Fig. 2b).

Mechanistically, the fMet-RQC pathway is distinguishable from canonical RQCs: disrupting canonical components (for example, Hel2, Dom34, and Ltn1) does not increase fMet-protein levels, whereas deleting fMet-RQC-specific factors (for example, Nip1 and Arf1) does^18^. Nonetheless, the fMet-RQC pathway likely cooperates with core RQC machinery — including the Dom34–Hbs1–Rli1 axis, the RQT complex, and the Rqc1/Rqc2/Ltn1 module — to eliminate stalled, fMet-bearing chains. The details of this interplay remain to be elucidated.

### Nip1 acts as Nt-fMet sensor on the 60S

Nip1, a core subunit of eIF3 that typically associates with the 40S ribosomal subunit during translation initiation, is repurposed during early elongation to recognize Nt-fMet on the 60S subunit^18,95^. Similar to other eIF3 subunits (for example, eIF3e and eIF3h) that monitor nascent-chain properties during elongation^96,97^, Nip1 senses emerging Nt-fMet and activates the fMet-RQC pathway independently of ribosome collisions or no-go decay/non-stop decay surveillance^18^.

By linking an initiation factor to co-translational quality control, Nip1 participates in a broader network of fMet-recognition systems. This includes PDF, which removes Nt-formyl groups from nascent chains^54^; FPRs (FPR1/FPR2), which mediate immune and stress signaling^20,21^; and fMet/N-recognins such as Psh1 and TRIM52, which target formylated proteins for proteasomal degradation^14,48^.

### ARF GTPases coordinate ribosome disassembly and stress-granule formation

Small GTPases of the ARF/ARL family, including yeast Arf1/Arf2 and mammalian ARF1, ARF3–5, are best known as GTP-regulated molecular switches involved in COPI/II vesicle trafficking, Golgi organization, lipid remodeling, and cytoskeletal dynamics^98–100^.

Recent studies expand their functional repertoire to include roles in RQC and stress granule (SG) assembly^18^. In yeast, GTP-bound Arf1/Arf2 associates with Nip1-bound 60S ribosomes during early elongation. Upon GTP hydrolysis, Arf1/2 stimulates Rli1-mediated ribosomal subunit dissociation. They also recruit Hsp104 to dissolve ribosome–nascent chain aggregates, whereas Hcr1 (eIF3j) stabilizes stalled 80S complexes^101^ and supports Rli1 loading during the Arf1/2 GTPase cycle^18^. Although these processes are characterized in yeast, direct mammalian evidence is not yet available.

Yeasts form SGs under near-freezing conditions as a protective response^102,103^. Arf1 has been implicated in cold tolerance^104^, although its mechanistic role was previously unclear. Recent evidence positions Arf1/Arf2 in fMet-RQC, in which they govern ribosome stalling and SG formation in response to cytosolic Nt-formylation. In *arf1*Δ cells, cytosolic *Sc*Fmt1 becomes toxic, as its detrimental effects outweigh the adaptive benefits of mitochondrial *Sc*Fmt1 during cold stress. This impairs fMet-RQC-dependent SG assembly and promotes aggregation of fMet-bearing polypeptides^18^.

Under acute cold stress, cytosolic *Sc*Fmt1 promotes fMet–tRNAi synthesis and activates fMet-RQC, thereby triggering SG assembly and repressing fMet-protein translation^18^. This response conserves ribosome and mRNA integrity while preserving^102,103^ (Fig. 2b). However, during prolonged cold, Arf1 levels decline and its binding to Nip1 weakens. As a result, fMet-RQC activity diminishes, leading to accumulation of toxic, aggregation-prone fMet-nascent chains and ribosome co-aggregation^18^ (Fig. 2b) — a phenomenon reminiscent of CAT-tail-induced aggregation seen in aging and neurodegeneration^77,78^.

Loss of Arf1/Arf2 allows continued fMet-protein synthesis^18^, which disrupts Nt-processing events (for example, Met excision and Nt-acetylation) and accelerates proteostasis collapse^1,2,11^. Although SG formation is often associated with eIF2α phosphorylation^105^, the precise roles of Nip1 and Arf1/2 in SG dynamics remain to be elucidated.

## The fMet/N-degron pathway

### Parallel evolution of Nt-surveillance

**Fig. 3:**
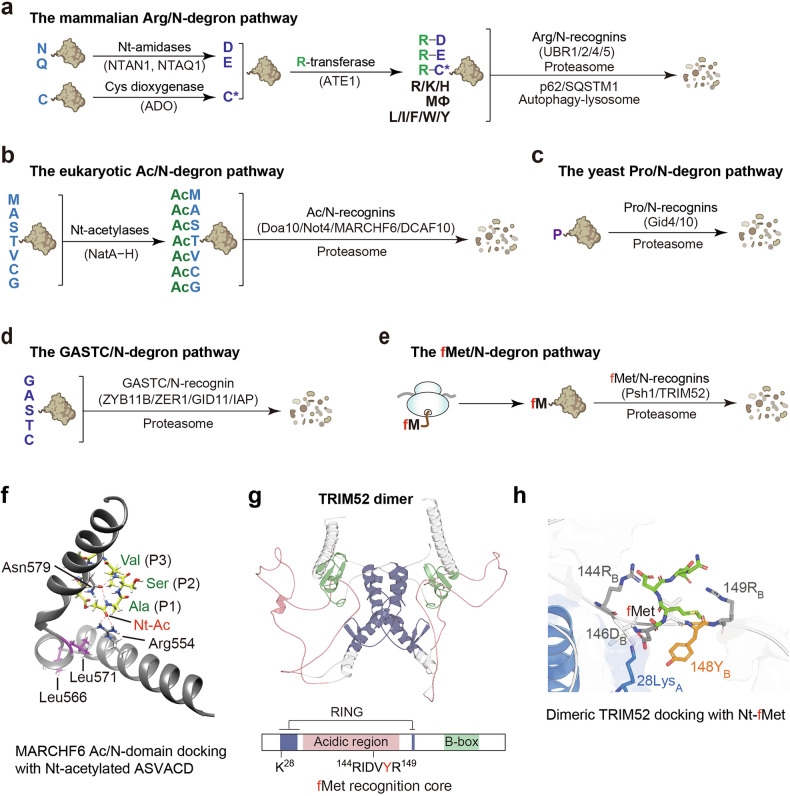
A unified framework of N-degron pathways and substrate recognition mechanisms. **a**–**e** Overview of eukaryotic N-degron pathways, classified by whether the Nt-residue is unmodified or bears an initiating modification and by the corresponding recognins^3,111,152^. **a** Mammalian Arg/N-degron pathway. Tertiary destabilizing residues Nt-Asn/Gln are deamidated by NTAN1/NTAQ1 to Nt-Asp/Glu, and Nt-Cys is oxidized by the dioxygenase ADO to Cys-sulfinic/sulfonic acid. These secondary residues are then arginylated by ATE1 to generate the primary destabilizing residue Nt-Arg. Primary destabilizing Nt-residues (Arg, Lys, His, Leu, Ile, Phe, Trp, Tyr, and Met-Φ (Met followed by a bulky hydrophobic at position 2)) are recognized by UBR1/2/4/5 (Arg/N-recognins), leading to polyubiquitylation and proteasomal degradation; arginylated clients can also be routed to the p62/SQSTM1-mediated autophagy–lysosome pathway. **b** The Ac/N-degron pathway. Nt-Met, Ser, Thr, Val, Ala, Cys, and Gly are co-translationally or post-translationally acetylated by NatA–NatH complexes, generating Ac/N-degrons. These are recognized by Ac/N-recognins, including Doa10 and Not4 in yeast and MARCHF6 and DCAF10 in mammals, triggering polyubiquitination and proteasomal degradation. **c** The yeast Pro/N-degron pathway. Nt-Pro is recognized by the GID E3 complex through interchangeable substrate receptors, including Gid4 (canonical) and Gid10 (conditional), driving polyubiquitination and proteasomal degradation. **d** The mammalian GASTC/N-degron pathway. Nt-Gly, Ala, Ser, Thr, and Cys are directly recognized by ZYG11B, ZER1, GID11, or IAP family members. **e** The fMet/N-degron pathway. Nt-fMet is specifically recognized by fMet/N-recognins, including Psh1 in yeast and TRIM52 in mammals, directing substrates to polyubiquitylation-mediated proteasomal degradation. **f** MARCHF6 Ac/N-degron recognition model. Structural model of the MARCHF6 Ac/N-recognin domain complexed with an Nt-acetylated peptide (Ac–Ala–Ser–Val–Ala–Val–Asp). Arg554 and Asn579 contact the Nt-acetyl group and position P1 to P3 of the peptide^150^. **g** TRIM52 architecture. Predicted dimeric organization of mammalian TRIM52, highlighting the RING domain, B-box, acidic region, and a putative fMet recognition core spanning residues 144–149 (ref. ^48^). **h** TRIM52–fMet docking. Docking of an Nt-fMet peptide into the TRIM52 dimer interface shows Tyr148 and nearby positively charged residues (Lys28, Arg144, and Arg149) coordinating the Nt-fMet, suggesting a unique recognition mechanism distinct from Ac/N-recognin.

Since the discovery of N-degrons in 1986, the chemical identity of a protein’s Nt-residue has been recognized as a key determinant of its half-life by directing proteins to distinct proteolytic pathways^3,106^. In eukaryotes, five major branches have emerged: the Arg/N-degron pathway (targeting unmodified Nt-Arg, Lys, His, Leu, Ile, Phe, Trp, Tyr, and Met-Φ (a hydrophobic residue)), Ac/N-degron pathway (acetylated Nt-residues), Pro/N-degron pathway (Nt-Pro), GASTC/N-degron pathway (Nt-Gly, Ala, Ser, Thr, and Cys), and the fMet/N-degron pathway (Nt-fMet). Together, these systems maintain proteostasis via the ubiquitin–proteasome and, in some contexts, autophagy–lysosome machineries^3,14,107–115^ (Fig. 3).

### Bacterial fMet/N-degron pathway

Bacterial translation initiates with fMet installed by FMT. As the nascent chain emerges from the 50S tunnel, PDF removes the formyl group, licensing Met aminopeptidase (MetAP), which preferentially excises Met when position 2 residue is small; PDF and MetAP compete at overlapping ribosomal sites near L22 (refs. ^9,116^). Genetics affirms this hierarchy: *fmt*Δ cells grow slowly yet survive^30^, whereas loss or inhibition of PDF is lethal in FMT-positive cells because MetAP cannot act on formylated N termini^11,117^; accordingly, *fmt*Δ *pdf*Δ double mutants resemble *fmt*Δ cells, underscoring the essential role of deformylation in Nt-maturation^118^. Despite robust co-translational deformylation, many nascent chains escape PDF owing to low PDF:ribosome stoichiometry (~2–3 µM versus ~30 µM), sequence-dependent kinetics, and early compaction that transiently buries the first ~10 residues^8,9^. Empirically, small DNA-encoded peptides frequently retain Nt-fMet — evidence that PDF escape is routine rather than exceptional^11,119^.

The notion that Nt-fMet can create a specific N-degron arose by analogy to the Ac/N-degron: a chemically analogous, position-specific modification that creates an N-degron^107^. Early hints came from chloroplast D2, in which blocking deformylation destabilized the protein^120^; later, bacterial reporters bearing the D2 N terminus showed formylation-dependent, PDF-sensitive turnover^9,14^. A ribosome-proximal fMet/N-degron was suggested: a recognin–protease module captures escaped Nt-fMet and initiates processive degradation^9^. The AAA⁺ protease FtsH was implicated in the turnover of YfgM, a membrane protein predicted to retain Nt-fMet, with Lon and ClpP-containing proteases as additional candidates of fMet/N-recognins^9,121^.

Conceptually, a tight PDF cloud processes most nascent chains co-translationally, whereas a looser recognin–protease cloud potentially intercepts PDF escapees co-translationally or post-translationally; trigger factor (engaging after ~100 residues) and SRP further shape the kinetic window by modulating early folding and Nt-exposure^9,58^. Functionally, the fMet/N-degron acts as front-line quality control under translational stress (for example, mistranslation and fidelity-lowering antibiotics), removing aberrant Nt-fMet species when PDF misses its window^9^. Nonetheless, key questions remain: identifying the bacterial fMet/N-recognin(s), mapping their native substrates, and elucidating how they coordinate with PDF in vivo^9,14^.

### The yeast fMet/N-degron pathway

Cytosolic translation in eukaryotes was long thought to start with unformylated Met, but N-terminomics revealed Nt-formylation on nuclear-encoded proteins in *S. cerevisiae*^14–17^. Low-level endogenous Nt-fMet also arises from incomplete mitochondrial import of the native *Sc*Fmt1 (refs. ^14,18^). Genetic and biochemical evidence established Nt-formylation as a degradation signal (fMet/N-degron)^14^. Particularly, co-expression of active *Ec*PDF removes Nt-fMet and stabilizes Nt-formylatable reporters, whereas an E3-ligase screen identified the tripartite motif (TRIM)-family E3 ligase Psh1 as the fMet/N-recognin^14^.

Psh1 directly binds Nt-fMet and, with its E2 partner Ubc3, mediates polyubiquitylation and proteasomal turnover; loss of Psh1 stabilizes these Nt-formylated proteins and sensitizes cells to proteotoxic stress, cold, and sodium azide^14^. Verified Psh1 clients with fMet/N-degrons include Cse4 (centromeric H3 variant), Pgd1 (mediator subunit), and Rps22a (40S subunit)^14^. Because Nt-formylation and Nt-acetylation are mutually exclusive, the fMet/N-degron branch likely complements the Ac/N-degron pathway: when Ac-CoA is limiting and Nt-acetylation wanes, Gcn2-driven *Sc*Fmt1 retention may elevate Nt-formylation so that Psh1-mediated clearance preserves proteostasis across fluctuating nutrient and temperature conditions^14^ (Fig. 3e).

### The mammalian fMet/N-degron pathway

TRIM proteins are a large family of RING-type E3 ligases with a conserved N-terminal tripartite architecture: a RING finger domain (for E2 engagement and ubiquitin transfer), one or two B-box domains (zinc-binding motifs for structural integrity and protein–protein interactions), and a coiled-coil domain (for oligomerization)^122^. Their C-terminal regions are more variable, incorporating domains such as PRY/SPRY, NHL, or PHD-BR that confer substrate specificity, particularly in immunity and cancer^123^.

In the yeast fMet/N-degron pathway, the E3 ligase Psh1 acts as fMet/N-recognin^14^. Structural similarity between yeast Psh1 and mammalian TRIMs^124^, along with evidence for a cytosolic pool of human MTFMT^47^, motivated the search for a mammalian fMet/N-degron pathway. This led to the identification of TRIM52 as a human fMet/N-recognin^48^.

TRIM52 functions as a dimer and recognizes Nt-fMet through an evolutionarily conserved acidic loop embedded within its bipartite (split) RING domain. Tyr148 is essential for substrate binding but dispensable for E3 catalysis; the Y148A mutation abolishes fMet recognition while preserving polyubiquitylation activity. Structural modeling and molecular-dynamics simulations suggest that the formyl group helps neutralize local electrostatic repulsion to stabilize the substrate–E3 interface^48^.

Endogenous Nt-formylated substrates of TRIM52 include TPD54 (a tumor-associated trafficking protein) and SPTAN1 (a spectrin-associated cytoskeletal protein), which are polyubiquitylated and subsequently degraded by the proteasome^48^. Loss of TRIM52 causes accumulation of Nt-formylated proteins and caspase-3-dependent apoptosis; both phenotypes are rescued by reintroducing wild-type TRIM52 or by enhancing deformylation, which reduces the pool of fMet-bearing substrates^48^. A cytosolic fraction of MTFMT catalyzes Nt-formylation of nuclear-encoded proteins, paralleling stress-induced relocalization of yeast Fmt1. Notably, human TRIM52 can functionally substitute for Psh1 in yeast, underscoring the evolutionary conservation of fMet/N-degron recognition^48^.

Beyond its fMet/N-degron activity, TRIM52 is implicated in antiviral defense, inflammation, genome maintenance, and cancer, acting through nuclear factor (NF)-κB, STAT3, and Wnt/β-catenin signaling^125–129^. It restricts Japanese encephalitis virus by promoting NS2A degradation and activates NF-κB to induce pro-inflammatory cytokines^130,131^. TRIM52 has also been proposed as a prognostic marker for sepsis^132^. Its levels are tightly controlled by proteolytic network; loss of regulation leads to accumulation of TOP2 (topoisomerase 2)–DNA adducts, cell-cycle arrest, and engagement of DNA-repair factors^133^. How TRIM52 integrates its role as the fMet/N-recognin with proteome surveillance, immune regulation, and genome integrity remains an open question^48^.

## fMet derivatives and physiological implications

### From bacterial signatures to endogenous signals

**Fig. 4:**
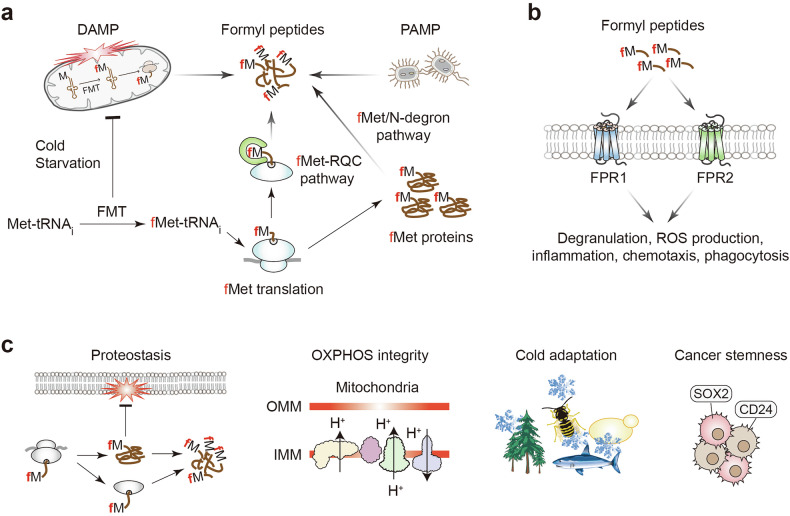
Sources, immune signaling, and physiological implications of cytosolic fMet translation and formyl peptides. **a** Multiple sources of formyl peptides. Mitochondrial stress or leakage releases fMet-containing peptides as DAMPs (damage-associated molecular patterns), whereas bacterial fMet peptides act as PAMPs (pathogen-associated molecular patterns). Independently, under cold stress or nutrient starvation in yeast and under certain cancer contexts, normally mitochondria-directed FMTs accumulate in the cytosol and produce fMet-tRNAi. Cytosolic initiation with fMet-tRNAi generates fMet-bearing nascent chains and proteins. These cytosolic fMet species are monitored by the fMet–ribosome quality control (RQC) pathway in yeast (a mammalian counterpart is yet to be defined) and by the fMet/N-degron pathway in both yeast and mammals, likely yielding short formylated peptides. **b** Formyl peptides from bacteria, mitochondria, or cytosolic translation act as potent immune cues by activating the G protein-coupled FPR1 and FPR2, triggering degranulation, reactive oxygen species (ROS) production, inflammatory signaling, chemotaxis, and phagocytosis. **c** Physiological and pathological implications of cytosolic fMet translation. Left panel: fMet impacts proteostasis by influencing protein synthesis, degradation, aggregation, and RQC and by competing with other essential Nt-modifications; it can also interfere with proper targeting to organelles or membranes. Middle-left panel: fMet regulation may intersect with mitochondrial oxidative phosphorylation (OXPHOS) and maintenance of the proton gradient. Middle-right panel: fMet pathways may modulate cold adaptation in poikilothermic organisms and in body extremities. Right panel: Nt-fMet suppresses cancer cell proliferation and stemness-like traits (for example, SOX2 and CD24), indicating broader pathophysiological relevance beyond immune signaling^46^. FMT, formyltransferase; FPR, formyl peptide receptor; IMM, inner mitochondrial membrane; OMM, outer mitochondrial membrane; tRNA, transfer RNA.

Formyl peptides arise from both exogenous and endogenous sources and have immune as well as broader physiological consequences (Fig. 4). N-formylated peptides were first linked to neutrophil chemotaxis when N-terminally blocked *E. coli* peptides were found to attract leukocytes; structure–activity studies subsequently identified formyl–Met–Leu–Phe (fMLF) as the prototypic ligand and led to the discovery of the neutrophil G protein-coupled receptor FPR1 (refs. ^134,135^). Once considered exclusively pathogen-associated molecular patterns, formyl peptides are now also recognized as damage-associated molecular patterns during sterile inflammation, and, in addition to bacterial and mitochondrial sources, endogenous stress-responsive ligands may arise through cytosolic fMet-protein synthesis, the fMet–RQC pathway, and the fMet/N-degron pathway^12,19,21,136^ (Fig. 4a).

FPR1 binds short formyl peptides with high affinity, whereas FPR2 is more permissive, accommodating broader range of ligands, including longer peptides, and can drive pro-inflammatory or anti-inflammatory signaling depending on ligand context; together, these receptors mediate chemotaxis, degranulation, reactive oxygen species production, inflammatory signaling, and phagocytosis in response to formyl peptides (Fig. 4b).

Notably, intact fMLF is rare in native samples and rapid deformylation or membrane sequestration limits extracellular release^54,58^. Beyond immune signaling, cytosolic fMet-linked pathways may have broader physiological and pathological consequences through effects on proteostasis, mitochondrial function, stress adaptation, and cancer-related phenotypes (Fig. 4c).

### Clinical associations and therapeutic angles

Circulating fMet rises sharply in acute inflammatory states, particularly septic shock, in which plasma levels track severity and mortality; levels exceed those in bacteremia without sepsis, implicating host-derived production. Elevated fMet and fMet-peptides is also reported in hypertension, severe COVID-19, systemic sclerosis, vasculitis, and rheumatoid arthritis, where it promotes neutrophil activation^137–144^. Population studies link high fMet to impaired mitochondrial translation, age-related disease, and increased all-cause mortality^141–144^. Neutralization strategies underscore pathogenicity: immobilized anti-formyl peptide antibodies restore neutrophil (polymorphonuclear leukocyte) function in sepsis models, and both peptide-specific and pan-specific anti-fMet antibodies are in development^15–17,145^. Although fMLF remains a canonical probe, its rarity in vivo highlights the need to target endogenous, stress-responsive ligands.

## Detection of fMet derivatives

### N-terminomics

Proteome-wide detection of fMet-bearing proteins has been challenging owing to their rare and transient nature — mainly caused by co-translational deformylation or proteolytic degradation^8,9,14^. Additional technical barriers include the chemically blocked α-amino group, which resists conventional Nt-labeling, and the small mass shift introduced by Nt-formylation (+27.9949 Da), which closely resembles that by dimethylation (+28.0313 Da). As a result, reliable detection of Nt-formylation has historically required ultrahigh-resolution mass spectrometry^146^.

**Fig. 5:**
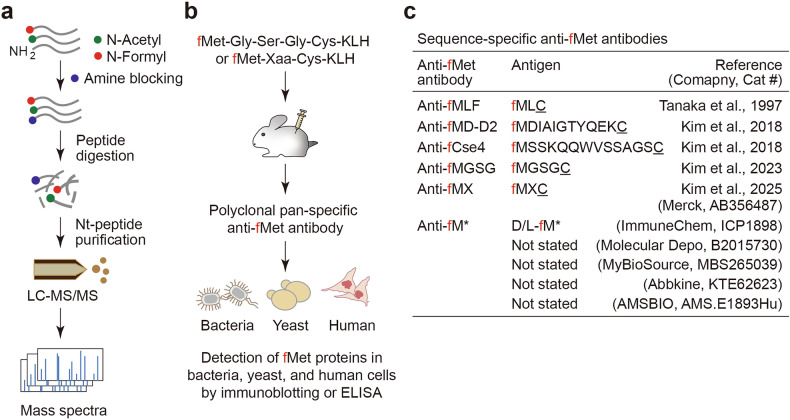
Experimental strategies for detecting N-terminally formylated peptides and proteins. **a** Workflow for Nt-proteomics analysis to detect formylated (fMet) and acetylated (AcMet) peptides. Proteins are subjected to chemical amine labeling to block free α-amines. After proteolytic digestion, Nt-peptides are enriched and analyzed by liquid chromatography tandem mass spectrometry (LC-MS/MS), allowing the identification of Nt-modifications based on mass spectra^13,148^. **b** Generation of polyclonal anti-fMet antibodies. Synthetic peptides containing Nt-fMet (for example, fMet–Gly–Ser–Gly–Cys: fMGSGC) or fMet–Xaa–Cys (fMXC, in which Xaa indicates any of the 20 standard amino acids) conjugated to keyhole limpet hemocyanin (KLH) are used to immunize rabbits, producing pan-specific anti-fMet antibodies. These antibodies enable immunodetection of fMet-containing proteins in bacteria, yeast, and human cells via immunoblotting or enzyme-linked immunmosorbent assay (ELISA)^15–17^. **c** Summary of anti-fMet antibodies. Sequence-specific anti-fMet antibodies recognize defined epitopes, including fMLC (anti-fMLF), fMDIAIGTYQEKC (anti-fMD-D2), fMSSKQQWVSSAGSC (anti-fCse4), fMGSGC (anti-fMGSG), and fMXC mixtures (X = any amino acid; anti-fMX; Merck, AB356487). C denotes the C-terminal cysteine added to conjugate the antigen peptides to carrier proteins. An anti-free fMet antibody (ImmuneChem, ICP1898) was raised against D-fMet or L-fMet conjugates. Although many commercial pan-fMet reagents are marketed for ELISA, in our hands these antibodies showed minimal reactivity toward N-terminally formylated peptides and proteins. By contrast, anti-fMXC gave the most reliable detection of Nt-formylated targets by immunoblotting and ELISA across bacteria, yeast, and mammalian cells. These reagents enable targeted detection and characterization of Nt-formylated species across systems.

Recent advances in N-terminomics have begun to overcome these obstacles. Platforms such as iNrich and its miniaturized variant, tipNrich, enable selective enrichment of Nt-peptides — including fMet-bearing species — from femtomole-scale inputs, without requiring large protein quantities^147,148^. These tools provide unprecedented sensitivity for detecting low-abundance Nt-formylated proteoforms implicated in stress responses and disease (Fig. 5a).

### Anti-fMet antibodies

Monoclonal anti-fMLF antibodies have been first used to detect free formyl peptides in enzyme-linked immunosorbent assays^149^. However, these antibodies exhibit limited affinity for formylated N termini embedded within polypeptides. As such, their application is generally restricted to specific formylated peptides and not suitable for general analysis.

To overcome these limitations, polyclonal pan-specific anti-fMet antibodies have been developed using both a defined peptide (fMet–Gly–Ser–Gly–Cys) and a synthetic peptide library featuring diverse second-position residues (fMet–Xaa–Cys; Xaa = any amino acid) as immunogens^14–17^. These antibodies exhibit broad sequence tolerance, high sensitivity, and cross-species reactivity, as validated by enzyme-linked immunosorbent assays and immunoblotting using synthetic peptides and a range of biological samples. They provide a scalable and cost-effective method for detecting Nt-formylated proteins across cytosolic, mitochondrial, and bacterial compartments (Fig. 5b).

Furthermore, many commercial anti-fMet reagents have been raised against free fMet haptens (Fig. 5c), which may not closely mimic the chemical context of Nt-formylated residues within peptide bonds. Consequently, their reactivity toward bona fide Nt-formylated proteins can be weak or variable (Fig. 5c). To ensure specificity, anti-fMet signals should be rigorously validated by: (i) competition assays using fMet peptides (not free fMet), (ii) genetic or pharmacological perturbation of FMT and PDF enzymes, and (iii) N-terminomic mass spectrometry of the same protein. Antibodies lacking these validation criteria should not be used to infer Nt-formylation in proteins.

### An integrated platform for Nt-formylation mapping

The integration of high-sensitivity N-terminomic profiling with pan-fMet antibody detection has established a versatile platform for mapping Nt-formylation across diverse species and stress conditions. These advances have enabled the discovery of stress-inducible cytosolic fMet-protein synthesis and its regulation by both the fMet-RQC and fMet/N-degron pathways^14,18,48^. Together, they open new avenues for exploring Nt-formylation as a regulatory mechanism in translation control, proteostasis maintenance, and its emerging roles in disease and therapy.

## Conclusions and future directions

Nt-formylation — once viewed as a prokaryotic relic — is now recognized as a conserved, stress-inducible cytosolic modification in eukaryotes. Under metabolic or environmental stress, Gcn2–*Sc*Fmt1-driven fMet initiation reshapes proteostasis by coordinating nascent-chain maturation, activating a dedicated fMet-RQC pathway and engaging the fMet/N-degron system for selective clearance^14,18^.

This program competes with other Nt-modifications and can redirect organelle and membrane targeting^1,2^. Its downstream effects intersect with mitochondrial metabolism (for example, OXPHOS), may facilitate cold adaptation in poikilotherms and peripheral tissues, and correlate with reduced proliferation and stemness-like traits in some cancers^14,18,46^.

Upstream regulation likely integrates ISR signaling and stress-dependent trafficking or activation of FMT. Partitioning of FMT between mitochondria and cytosol, combined with ISR input, likely defines quantitative thresholds for cytosolic Nt-formylation. By tuning the pool of fMet–tRNAᵢ in a tissue-specific and stress-specific manner, this axis potentially connects nutrient-sensing networks (for example, mTOR and AMPK) to translation initiation chemistry and broader homeostatic programs.

Mechanistically, the fMet-RQC pathway is established in yeast, and several core components (eIF3C, eIF3J, ARF GTPases, and ABCE1) are conserved, motivating biochemical reconstitution and structural analysis in mammals^18^. Key priorities include: (i) elucidating delivery routes for fMet–tRNAᵢ (eIF2D–DENR/MCTS1 versus eIF5B), (ii) identifying nascent-chain and ribosomal tunnel features that enforce the ≈codon-41 stall, (iii) characterizing mammalian counterparts of eIF3C and eIF3J, and (iv) resolving ARF/ARL–ABCE1 mechanics during ribosome splitting. Determining whether a cytosolic deformylase exists in eukaryotes and how its ISR/mTOR-regulated kinetics compete with MetAPs and Nt-acetyltransferases will clarify proteome-wide partitioning throughout maturation, fMet-RQC, and fMet/N-degron fates, as well as crosstalk among Nt-modifications.

A comprehensive atlas is now essential. Proteome-scale mapping of Nt-formylated proteins and formyl-peptides across cell types, tissues, and stress will define the eukaryotic substrate landscape and constrain models of specificity. Because direct detection is limited by lability and scarcity, progress will rely on sensitive formyl-preserving peptidomics (for example, short-peptide enrichment and derivatization/ion-mobility strategies), orthogonal validation (fit-for-purpose anti-fMet reagents and genetic/chemical perturbation of FMT, PDF, and TRIM52), and rigorous attribution to separate cytosolic from mitochondrial/bacterial products. Extending these pipelines to in vivo system will evaluate circulating formyl-peptides/proteins as biomarkers of cellular stress and disease states.

Across kingdoms, E3 ligases decode initiation chemistry to couple synthesis with degradation. In mammals, TRIM52 recognizes Nt-fMet and eliminates cytotoxic fMet-bearing proteins through proteasomal elimination, thereby maintaining proteostasis^48^. Defining the grammar of the mammalian fMet/N-degron — including substrate motifs, the endogenous substrateome, regulatory inputs, and integration with innate immunity and stress signaling — will establish the organizing principles of this pathway and clarify the conservation of fMet/N-degron pathway in multicellular eukaryotes.

The Nt-formylation axis is tractable: engineer mtPDFs for cytosolic activity; tune the balance between Nt-formylation and deformylation to regulate protein stability; modulate TRIM52 to reinforce proteostasis; and neutralize formyl peptides to temper FPR-driven inflammation. Systematic mapping of Nt-formylation dynamics across tissues and disease states promises to refine core principles of translational control and stress adaptation, while advancing diagnostics and interventions in immunity, aging, neurodegeneration, ischemia–reperfusion injury, and oncology.

## Data Availability

Data sharing is not applicable to this article, as no data sets were generated or analyzed during the current study.

## References

[1] Oye, H., Lundekvam, M., Caiella, A., Hellesvik, M. & Arnesen, T. Protein N-terminal modifications: molecular machineries and biological implications. *Trends Biochem Sci* **50**, 290–310 (2025). 39837675 10.1016/j.tibs.2024.12.012

[2] Meinnel, T. & Giglione, C. N-terminal modifications, the associated processing machinery, and their evolution in plastid-containing organisms. *J Exp Bot* **73**, 6013–6033 (2022). 35768189 10.1093/jxb/erac290

[3] Varshavsky, A. N-degron pathways. *Proc Natl Acad Sci USA* **121**, e2408697121 (2024). 39264755 10.1073/pnas.2408697121 PMC11441550

[4] Adams, J. M. & Capecchi, M. R. *N*-formylmethionyl-sRNA as the initiator of protein synthesis. *Proc Natl Acad Sci USA* **55**, 147–155 (1966). 5328638 10.1073/pnas.55.1.147 PMC285768

[5] Laursen, B. S., Sorensen, H. P., Mortensen, K. K. & Sperling-Petersen, H. U. Initiation of protein synthesis in bacteria. *Microbiol Mol Biol Rev* **69**, 101–123 (2005). 15755955 10.1128/MMBR.69.1.101-123.2005 PMC1082788

[6] Webster, M. W. Initiation of translation in bacteria and chloroplasts. *J Mol Biol* **437**, 169137 (2025). 40221131 10.1016/j.jmb.2025.169137

[7] Lahry, K., Datta, M. & Varshney, U. Genetic analysis of translation initiation in bacteria: an initiator tRNA-centric view. *Mol Microbiol* **122**, 772–788 (2024). 38410838 10.1111/mmi.15243

[8] Bogeholz, L. A. K., Mercier, E., Wintermeyer, W. & Rodnina, M. V. Kinetic control of nascent protein biogenesis by peptide deformylase. *Sci Rep* **11**, 24457 (2021). 34961771 10.1038/s41598-021-03969-3 PMC8712518

[9] Piatkov, K. I., Vu, T. M., Hwang, C. S. & Varshavsky, A. Formyl-methionine as a degradation signal at the N-termini of bacterial proteins. *Microbial Cell* **2**, 376–393 (2015). 26866044 10.15698/mic2015.10.231 PMC4745127

[10] Hinttala, R. et al. An N-terminal formyl methionine on COX 1 is required for the assembly of cytochrome *c* oxidase. *Hum Mol Genet* **24**, 4103–4113 (2015). 25911677 10.1093/hmg/ddv149 PMC4476453

[11] Yang, C. I. et al. System-wide analyses reveal essential roles of N-terminal protein modification in bacterial membrane integrity. *iScience* **25**, 104756 (2022). 35942092 10.1016/j.isci.2022.104756 PMC9356101

[12] Lee, C. S., Kim, D. & Hwang, C. S. Where does *N*-formylmethionine come from? What for? Where is it going? What is the origin of *N*-formylmethionine in eukaryotic cells? *Mol Cells* **45**, 109–111 (2022). 35288488 10.14348/molcells.2021.5040 PMC8926868

[13] Kaushal, P. & Lee, C. N-terminomics — its past and recent advancements. *J Proteom* **233**, 104089 (2021). 10.1016/j.jprot.2020.104089 33359939

[14] Kim, J. M. et al. Formyl-methionine as an N-degron of a eukaryotic N-end rule pathway. *Science* **362**, eaat0174 (2018). 30409808 10.1126/science.aat0174 PMC6551516

[15] Kim, D. et al. Detection of Nalpha-terminally formylated native proteins by a pan-*N*-formyl methionine-specific antibody. *J Biol Chem* **299**, 104652 (2023). 36990220 10.1016/j.jbc.2023.104652 PMC10164907

[16] Kim, D., Park, K. S. & Hwang, C. S. Development of an enhanced anti-pan-*N*-formylmethionine-specific antibody. *Biotechniques* **77**, 46–55 (2025). 39973362 10.1080/07366205.2025.2467583

[17] Kim, D., Baek, S., Lee, C. S., Lee, E. J. & Hwang, C. S. A pan-*N*-formylmethionine-specific antibody as a tool for analyzing Nalpha-terminal formylation. *Methods Enzymol* **718**, 185–214 (2025). 40887158 10.1016/bs.mie.2025.06.001

[18] Lee, C. S. et al. Formyl-methionine-mediated eukaryotic ribosome quality control pathway for cold adaptation. *Mol Cell* **85**, 602–619.e16 (2025). 39721582 10.1016/j.molcel.2024.11.035

[19] Lee, C. S. & Hwang, C. S. Cytosolic fMet-protein synthesis as source of endogenous ligands for formyl peptide receptors. *Bioessays* **47**, e70074 (2025). 41017204 10.1002/bies.70074

[20] Dahlgren, C. & Forsman, H. What is the potential of formyl peptide receptor 1 (FPR1) as a therapeutic target in human disease? *Exp Opin Ther Targets* **29**, 409–413 (2025). 10.1080/14728222.2025.2512525 40437796

[21] Napolitano, F. & Montuori, N. The *N*-formyl peptide receptors: much more than chemoattractant receptors. Relevance in health and disease. *Front Immunol* **16**, 1568629 (2025). 40103822 10.3389/fimmu.2025.1568629 PMC11913705

[22] Brischigliaro, M., Ahn, A., Hong, S., Fontanesi, F. & Barrientos, A. Emerging mechanisms of human mitochondrial translation regulation. *Trends Biochem Sci* **50**, 566–584 (2025). 40221217 10.1016/j.tibs.2025.03.007 PMC12227304

[23] Baleva, M. V., Piunova, U. E., Chicherin, I. V., Levitskii, S. A. & Kamenski, P. A. Diversity and evolution of mitochondrial translation apparatus. *Biochemistry* **88**, 1832–1843 (2023). 38105202 10.1134/S0006297923110135

[24] Jiang, T., Zhou, X., Taghizadeh, K., Dong, M. & Dedon, P. C. N-formylation of lysine in histone proteins as a secondary modification arising from oxidative DNA damage. *Proc Natl Acad Sci USA* **104**, 60–65 (2007). 17190813 10.1073/pnas.0606775103 PMC1765477

[25] Edrissi, B., Taghizadeh, K. & Dedon, P. C. Quantitative analysis of histone modifications: formaldehyde is a source of pathological *N*(6)-formyllysine that is refractory to histone deacetylases. *PLoS Genet* **9**, e1003328 (2013). 23468656 10.1371/journal.pgen.1003328 PMC3585032

[26] Sah, S. & Varshney, U. Methionyl-tRNA formyltransferase utilizes 10-formyldihydrofolate as an alternative substrate and impacts antifolate drug action. *Microbiology* **169**, 001297 (2023). 10.1099/mic.0.001297 PMC10197868 36745551

[27] Schmitt, E., Panvert, M., Blanquet, S. & Mechulam, Y. Crystal structure of methionyl-tRNAfMet transformylase complexed with the initiator formyl-methionyl-tRNAfMet. *EMBO J* **17**, 6819–6826 (1998). 9843487 10.1093/emboj/17.23.6819 PMC1171029

[28] Ramesh, V., Gite, S. & RajBhandary, U. L. Functional interaction of an arginine conserved in the sixteen amino acid insertion module of *Escherichia coli* methionyl-tRNA formyltransferase with determinants for formylation in the initiator tRNA. *Biochemistry* **37**, 15925–15932 (1998). 9843398 10.1021/bi981873x

[29] Lee, C. P., Seong, B. L. & RajBhandary, U. L. Structural and sequence elements important for recognition of *Escherichia coli* formylmethionine tRNA by methionyl-tRNA transformylase are clustered in the acceptor stem. *J Biol Chem* **266**, 18012–18017 (1991). 1917939

[30] Guillon, J. M., Mechulam, Y., Schmitter, J. M., Blanquet, S. & Fayat, G. Disruption of the gene for Met-tRNA(fMet) formyltransferase severely impairs growth of *Escherichia coli*. *J Bacteriol* **174**, 4294–4301 (1992). 1624424 10.1128/jb.174.13.4294-4301.1992 PMC206212

[31] Ramesh, V., Kohrer, C. & RajBhandary, U. L. Expression of *Escherichia coli* methionyl-tRNA formyltransferase in *Saccharomyces cerevisiae* leads to formylation of the cytoplasmic initiator tRNA and possibly to initiation of protein synthesis with formylmethionine. *Mol Cell Biol* **22**, 5434–5442 (2002). 12101237 10.1128/MCB.22.15.5434-5442.2002 PMC133937

[32] Halbreich, A. & Rabinowitz, M. Isolation of *Saccharomyces cerevisiae* mitochondrial formyltetrahydrofolic acid:methionyl-tRNA transformylase and the hybridization of mitochondrial fMet-tRNA with mitochondrial DNA. *Proc Natl Acad Sci USA* **68**, 294–298 (1971). 5277072 10.1073/pnas.68.2.294 PMC388921

[33] Takeuchi, N. et al. Recognition of tRNAs by methionyl-tRNA transformylase from mammalian mitochondria. *J Biol Chem* **276**, 20064–20068 (2001). 11274157 10.1074/jbc.M101007200

[34] Tucker, E. J. et al. Mutations in MTFMT underlie a human disorder of formylation causing impaired mitochondrial translation. *Cell Metab* **14**, 428–434 (2011). 21907147 10.1016/j.cmet.2011.07.010 PMC3486727

[35] Hayhurst, H. et al. Leigh syndrome caused by mutations in MTFMT is associated with a better prognosis. *Ann Clin Transl Neurol* **6**, 515–524 (2019). 30911575 10.1002/acn3.725 PMC6414492

[36] Giege, R. & Eriani, G. The tRNA identity landscape for aminoacylation and beyond. *Nucleic Acids Res* **51**, 1528–1570 (2023). 36744444 10.1093/nar/gkad007 PMC9976931

[37] Basu, R. S., Sherman, M. B. & Gagnon, M. G. Compact IF2 allows initiator tRNA accommodation into the P site and gates the ribosome to elongation. *Nat Commun* **13**, 3388 (2022). 35697706 10.1038/s41467-022-31129-2 PMC9192638

[38] Lahry, K., Gopal, A., Sah, S., Shah, R. A. & Varshney, U. Metabolic flux of N(10)-formyltetrahydrofolate plays a critical role in the fidelity of translation initiation in *Escherichia coli*. *J Mol Biol* **432**, 5473–5488 (2020). 32795532 10.1016/j.jmb.2020.08.003

[39] Spencer, A. C. & Spremulli, L. L. Interaction of mitochondrial initiation factor 2 with mitochondrial fMet-tRNA. *Nucleic Acids Res* **32**, 5464–5470 (2004). 15477394 10.1093/nar/gkh886 PMC524296

[40] Antolinez-Fernandez, A., Esteban-Ramos, P., Fernandez-Moreno, M. A. & Clemente, P. Molecular pathways in mitochondrial disorders due to a defective mitochondrial protein synthesis. *Front Cell Dev Biol* **12**, 1410245 (2024). 38855161 10.3389/fcell.2024.1410245 PMC11157125

[41] Schmitt, E. et al. Recent advances in archaeal translation initiation. *Front Microbiol* **11**, 584152 (2020). 33072057 10.3389/fmicb.2020.584152 PMC7531240

[42] Kazan, R. et al. Role of aIF5B in archaeal translation initiation. *Nucleic Acids Res* **50**, 6532–6548 (2022). 35694843 10.1093/nar/gkac490 PMC9226500

[43] Grosjean, H. et al. Predicting the minimal translation apparatus: lessons from the reductive evolution of mollicutes. *PLoS Genet* **10**, e1004363 (2014). 24809820 10.1371/journal.pgen.1004363 PMC4014445

[44] McCutcheon, J. P., Garber, A. I., Spencer, N. & Warren, J. M. How do bacterial endosymbionts work with so few genes? *PLoS Biol* **22**, e3002577 (2024). 38626194 10.1371/journal.pbio.3002577 PMC11020763

[45] Husnik, F. et al. Horizontal gene transfer from diverse bacteria to an insect genome enables a tripartite nested mealybug symbiosis. *Cell* **153**, 1567–1578 (2013). 23791183 10.1016/j.cell.2013.05.040

[46] Kim, D., Lee, J., Seok, O. H., Lee, Y. & Hwang, C. S. Cytosolic N-terminal formyl-methionine deformylation derives cancer stem cell features and tumor progression. *Sci Rep* **14**, 14900 (2024). 38942903 10.1038/s41598-024-65701-1 PMC11213908

[47] Thul, P. J. et al. A subcellular map of the human proteome. *Science* **356**, eaal3321 (2017). 10.1126/science.aal3321 28495876

[48] Kim, D. et al. TRIM52 ubiquitin ligase acts as a key recognition component of the mammalian fMet/N-degron pathway. *J Mol Biol* **437**, 169453 (2025). 10.1016/j.jmb.2025.169453 40976346

[49] Nanjaraj Urs, A. N., Kim, L. & Zaher, H. S. Insights into the role of collided ribosomes during the activation of the integrated stress response. *Biochem Soc Trans* **53**, 615–626 (2025). 40440025 10.1042/BST20253034 PMC12224898

[50] Zhou, C. et al. GCN1 couples GCN2 to ribosomal state to initiate amino acid response pathway signaling. *Science* **390**, eads8728 (2025). 41037622 10.1126/science.ads8728

[51] Gaikwad, S., Ghobakhlou, F., Zhang, H. & Hinnebusch, A. G. Yeast eIF2A has a minimal role in translation initiation and uORF-mediated translational control in vivo. *eL**ife* **12**, RP92916 (2024). 10.7554/eLife.92916 PMC10945734 38266075

[52] Roiuk, M., Neff, M. & Teleman, A. A. Human eIF2A has a minimal role in translation initiation and in uORF-mediated translational control in HeLa cells. *eLife* **14**, RP105311(2025). 10.7554/eLife.105311 PMC12221301 40600802

[53] Grove, D. J., Russell, P. J. & Kearse, M. G. To initiate or not to initiate: a critical assessment of eIF2A, eIF2D, and MCT-1.DENR to deliver initiator tRNA to ribosomes. *Wiley Interdiscip Rev RNA* **15**, e1833 (2024). 38433101 10.1002/wrna.1833 PMC11260288

[54] Bingel-Erlenmeyer, R. et al. A peptide deformylase-ribosome complex reveals mechanism of nascent chain processing. *Nature* **452**, 108–111 (2008). 18288106 10.1038/nature06683

[55] McGrath, H., Cernekova, M. & Kolar, M. H. Binding of the peptide deformylase on the ribosome surface modulates the exit tunnel interior. *Biophys J* **121**, 4443–4451 (2022). 36335428 10.1016/j.bpj.2022.11.004 PMC9748369

[56] Becker, A. et al. Iron center, substrate recognition and mechanism of peptide deformylase. *Nat Struct Biol* **5**, 1053–1058 (1998). 9846875 10.1038/4162

[57] Ragusa, S., Mouchet, P., Lazennec, C., Dive, V. & Meinnel, T. Substrate recognition and selectivity of peptide deformylase. Similarities and differences with metzincins and thermolysin. *J Mol Biol* **289**, 1445–1457 (1999). 10373378 10.1006/jmbi.1999.2832

[58] Ranjan, A., Mercier, E., Bhatt, A. & Wintermeyer, W. Signal recognition particle prevents N-terminal processing of bacterial membrane proteins. *Nat Commun* **8**, 15562 (2017). 28516953 10.1038/ncomms15562 PMC5454389

[59] Meinnel, T. & Blanquet, S. Evidence that peptide deformylase and methionyl-tRNA(fMet) formyltransferase are encoded within the same operon in *Escherichia coli*. *J Bacteriol* **175**, 7737–7740 (1993). 8244948 10.1128/jb.175.23.7737-7740.1993 PMC206938

[60] Giglione, C., Serero, A., Pierre, M., Boisson, B. & Meinnel, T. Identification of eukaryotic peptide deformylases reveals universality of N-terminal protein processing mechanisms. *EMBO J* **19**, 5916–5929 (2000). 11060042 10.1093/emboj/19.21.5916 PMC305796

[61] Serero, A., Giglione, C., Sardini, A., Martinez-Sanz, J. & Meinnel, T. An unusual peptide deformylase features in the human mitochondrial N-terminal methionine excision pathway. *J Biol Chem* **278**, 52953–52963 (2003). 14532271 10.1074/jbc.M309770200

[62] Karnkowska, A. et al. A eukaryote without a mitochondrial organelle. *Curr Biol* **26**, 1274–1284 (2016). 27185558 10.1016/j.cub.2016.03.053

[63] Frank, J. A. et al. Structure and function of a cyanophage-encoded peptide deformylase. *ISME J* **7**, 1150–1160 (2013). 23407310 10.1038/ismej.2013.4 PMC3660681

[64] Grzela, R. et al. The C-terminal residue of phage Vp16 PDF, the smallest peptide deformylase, acts as an offset element locking the active conformation. *Sci Rep* **7**, 11041 (2017). 28887476 10.1038/s41598-017-11329-3 PMC5591237

[65] Escobar-Alvarez, S. et al. Inhibition of human peptide deformylase disrupts mitochondrial function. *Mol Cell Biol* **30**, 5099–5109 (2010). 20805355 10.1128/MCB.00469-10 PMC2953058

[66] Randhawa, H. et al. Overexpression of peptide deformylase in breast, colon, and lung cancers. *BMC Cancer* **13**, 321 (2013). 23815882 10.1186/1471-2407-13-321 PMC3722014

[67] Saravanakumar, K. et al. Eradication of *Helicobacter pylori* through the inhibition of urease and peptide deformylase: Computational and biological studies. *Microb Pathog* **128**, 236–244 (2019). 30611769 10.1016/j.micpath.2019.01.001

[68] Costa-Martini, J. H., Adams, E. E. & Johnston, C. W. Chemotype- and target-driven genome mining for a new natural product inhibitor of bacterial peptide deformylase. *J Am Chem Soc* **147**, 21400–21407 (2025). 40493376 10.1021/jacs.4c17876 PMC12203648

[69] Giglione, C. & Meinnel, T. Peptide deformylase as an emerging target for antiparasitic agents. *Expert Opin Ther Targets* **5**, 41–57 (2001). 15992167 10.1517/14728222.5.1.41

[70] Guilloteau, J. P. et al. The crystal structures of four peptide deformylases bound to the antibiotic actinonin reveal two distinct types: a platform for the structure-based design of antibacterial agents. *J Mol Biol* **320**, 951–962 (2002). 12126617 10.1016/s0022-2836(02)00549-1

[71] Escobar-Alvarez, S. et al. Structure and activity of human mitochondrial peptide deformylase, a novel cancer target. *J Mol Biol* **387**, 1211–1228 (2009). 19236878 10.1016/j.jmb.2009.02.032 PMC2782631

[72] Doma, M. K. & Parker, R. Endonucleolytic cleavage of eukaryotic mRNAs with stalls in translation elongation. *Nature* **440**, 561–564 (2006). 16554824 10.1038/nature04530 PMC1839849

[73] Bengtson, M. H. & Joazeiro, C. A. Role of a ribosome-associated E3 ubiquitin ligase in protein quality control. *Nature* **467**, 470–473 (2010). 20835226 10.1038/nature09371 PMC2988496

[74] Brandman, O. et al. A ribosome-bound quality control complex triggers degradation of nascent peptides and signals translation stress. *Cell* **151**, 1042–1054 (2012). 23178123 10.1016/j.cell.2012.10.044 PMC3534965

[75] Guydosh, N. R. & Green, R. Dom34 rescues ribosomes in 3’ untranslated regions. *Cell* **156**, 950–962 (2014). 24581494 10.1016/j.cell.2014.02.006 PMC4022138

[76] Kuroha, K., Zinoviev, A., Hellen, C. U. T. & Pestova, T. V. Release of ubiquitinated and non-ubiquitinated nascent chains from stalled mammalian ribosomal complexes by ANKZF1 and Ptrh1. *Mol Cell* **72**, 286–302.e8 (2018). 30244831 10.1016/j.molcel.2018.08.022 PMC6344051

[77] Stein, K. C., Morales-Polanco, F., van der Lienden, J., Rainbolt, T. K. & Frydman, J. Ageing exacerbates ribosome pausing to disrupt cotranslational proteostasis. *Nature* **601**, 637–642 (2022). 35046576 10.1038/s41586-021-04295-4 PMC8918044

[78] McGirr, T., Onar, O. & Jafarnejad, S. M. Dysregulated ribosome quality control in human diseases. *FEBS J* **292**, 936–959 (2025). 38949989 10.1111/febs.17217 PMC11880988

[79] Filbeck, S., Cerullo, F., Pfeffer, S. & Joazeiro, C. A. P. Ribosome-associated quality-control mechanisms from bacteria to humans. *Mol Cell* **82**, 1451–1466 (2022). 35452614 10.1016/j.molcel.2022.03.038 PMC9034055

[80] Inada, T. & Beckmann, R. Mechanisms of translation-coupled quality control. *J Mol Biol* **436**, 168496 (2024). 38365086 10.1016/j.jmb.2024.168496

[81] Uematsu, S. & Qian, S. B. Interpreting ribosome dynamics during mRNA translation. *J Biol Chem* **301**, 110469 (2025). 40651611 10.1016/j.jbc.2025.110469 PMC12340396

[82] Lee, J. et al. Pelota-mediated ribosome-associated quality control counteracts aging and age-associated pathologies across species. *Proc Natl Acad Sci USA* **122**, e2505217122 (2025). 40758887 10.1073/pnas.2505217122 PMC12358915

[83] Monaghan, L., Longman, D. & Caceres, J. F. Translation-coupled mRNA quality control mechanisms. *EMBO J* **42**, e114378 (2023). 37605642 10.15252/embj.2023114378 PMC10548175

[84] Sundaramoorthy, E. et al. ZNF598 and RACK1 regulate mammalian ribosome-associated quality control function by mediating regulatory 40S ribosomal ubiquitylation. *Mol Cell* **65**, 751–760.e4 (2017). 28132843 10.1016/j.molcel.2016.12.026 PMC5321136

[85] Juszkiewicz, S. et al. ZNF598 is a quality control sensor of collided ribosomes. *Mol Cell* **72**, 469–481.e7 (2018). 30293783 10.1016/j.molcel.2018.08.037 PMC6224477

[86] Shoemaker, C. J., Eyler, D. E. & Green, R. Dom34:Hbs1 promotes subunit dissociation and peptidyl-tRNA drop-off to initiate no-go decay. *Science* **330**, 369–372 (2010). 20947765 10.1126/science.1192430 PMC4022135

[87] Becker, T. et al. Structural basis of highly conserved ribosome recycling in eukaryotes and archaea. *Nature* **482**, 501–506 (2012). 22358840 10.1038/nature10829 PMC6878762

[88] Ikeuchi, K. et al. Collided ribosomes form a unique structural interface to induce Hel2-driven quality control pathways. *EMBO J* **38**, e100276 (2019). 10.15252/embj.2018100276 PMC6396155 30609991

[89] Ford, P. W., Narasimhan, M. & Bennett, E. J. Ubiquitin-dependent translation control mechanisms: degradation and beyond. *Cell Rep* **43**, 115050 (2024). 39661518 10.1016/j.celrep.2024.115050 PMC11756260

[90] Shen, P. S. et al. Protein synthesis. Rqc2p and 60S ribosomal subunits mediate mRNA-independent elongation of nascent chains. *Science* **347**, 75–78 (2015). 25554787 10.1126/science.1259724 PMC4451101

[91] Verma, R. et al. Vms1 and ANKZF1 peptidyl-tRNA hydrolases release nascent chains from stalled ribosomes. *Nature* **557**, 446–451 (2018). 29632312 10.1038/s41586-018-0022-5 PMC6226276

[92] Lin, H. C. et al. C-terminal end-directed protein elimination by CRL2 ubiquitin ligases. *Mol Cell* **70**, 602–613.e3 (2018). 29775578 10.1016/j.molcel.2018.04.006 PMC6145449

[93] Koren, I. et al. The eukaryotic proteome is shaped by E3 ubiquitin ligases targeting C-terminal degrons. *Cell* **173**, 1622–1635.e14 (2018). 29779948 10.1016/j.cell.2018.04.028 PMC6003881

[94] Patil, P. R. et al. Mechanism and evolutionary origins of alanine-tail C-degron recognition by E3 ligases Pirh2 and CRL2-KLHDC10. *Cell Rep* **42**, 113100 (2023). 37676773 10.1016/j.celrep.2023.113100 PMC10591846

[95] Brito Querido, J., Diaz-Lopez, I. & Ramakrishnan, V. The molecular basis of translation initiation and its regulation in eukaryotes. *Nat Rev Mol Cell Biol* **25**, 168–186 (2024). 38052923 10.1038/s41580-023-00624-9

[96] Lin, Y. et al. eIF3 Associates with 80S ribosomes to promote translation elongation, mitochondrial homeostasis, and muscle health. *Mol Cell* **79**, 575–587.e7 (2020). 32589965 10.1016/j.molcel.2020.06.003

[97] Wolf, D. A., Lin, Y., Duan, H. & Cheng, Y. eIF-Three to Tango: emerging functions of translation initiation factor eIF3 in protein synthesis and disease. *J Mol Cell Biol* **12**, 403–409 (2020). 32279082 10.1093/jmcb/mjaa018 PMC7333474

[98] Jackson, C. L., Menetrey, J., Sivia, M., Dacks, J. B. & Elias, M. An evolutionary perspective on Arf family GTPases. *Curr Opin Cell Biol* **85**, 102268 (2023). 39491309 10.1016/j.ceb.2023.102268

[99] Li, F. L. & Guan, K. L. The Arf family GTPases: regulation of vesicle biogenesis and beyond. *Bioessays* **45**, e2200214 (2023). 36998106 10.1002/bies.202200214 PMC10282109

[100] Dejgaard, S. Y. & Presley, J. F. Arfs on the Golgi: four conductors, one orchestra. *Front Mol Biosci* **12**, 1612531 (2025). 40821699 10.3389/fmolb.2025.1612531 PMC12350278

[101] Young, D. J. & Guydosh, N. R. Hcr1/eIF3j is a 60S ribosomal subunit recycling accessory factor in vivo. *Cell Rep* **28**, 39–50 e34 (2019). 31269449 10.1016/j.celrep.2019.05.111 PMC6661068

[102] Grousl, T., Vojtova, J., Hasek, J. & Vomastek, T. Yeast stress granules at a glance. *Yeast* **39**, 247–261 (2022). 34791685 10.1002/yea.3681

[103] Hofmann, S., Cherkasova, V., Bankhead, P., Bukau, B. & Stoecklin, G. Translation suppression promotes stress granule formation and cell survival in response to cold shock. *Mol Biol Cell* **23**, 3786–3800 (2012). 22875991 10.1091/mbc.E12-04-0296 PMC3459856

[104] Stearns, T., Willingham, M. C., Botstein, D. & Kahn, R. A. ADP-ribosylation factor is functionally and physically associated with the Golgi complex. *Proc Natl Acad Sci USA* **87**, 1238–1242 (1990). 2105501 10.1073/pnas.87.3.1238 PMC53446

[105] Hofmann, S., Kedersha, N., Anderson, P. & Ivanov, P. Molecular mechanisms of stress granule assembly and disassembly. *Biochim Biophys Acta Mol Cell Res* **1868**, 118876 (2021). 33007331 10.1016/j.bbamcr.2020.118876 PMC7769147

[106] Bachmair, A., Finley, D. & Varshavsky, A. In vivo half-life of a protein is a function of its amino-terminal residue. *Science* **234**, 179–186 (1986). 3018930 10.1126/science.3018930

[107] Hwang, C. S., Shemorry, A. & Varshavsky, A. N-terminal acetylation of cellular proteins creates specific degradation signals. *Science* **327**, 973–977 (2010). 20110468 10.1126/science.1183147 PMC4259118

[108] Chen, S. J., Wu, X., Wadas, B., Oh, J. H. & Varshavsky, A. An N-end rule pathway that recognizes proline and destroys gluconeogenic enzymes. *Science* **355**, eaal3655 (2017). 28126757 10.1126/science.aal3655 PMC5457285

[109] Timms, R. T. et al. A glycine-specific N-degron pathway mediates the quality control of protein N-myristoylation. *Science* **365**, eaaw4912 (2019). 10.1126/science.aaw4912 PMC7090375 31273098

[110] Yang, J. & Hwang, C. S. Nalpha-terminal acetylation meets ferroptosis via N-degron pathway. *Mol Cells* **47**, 100160 (2024). 39577745 10.1016/j.mocell.2024.100160 PMC11697038

[111] Heo, A. J., Ji, C. H. & Kwon, Y. T. The Cys/N-degron pathway in the ubiquitin-proteasome system and autophagy. *Trends Cell Biol* **33**, 247–259 (2023). 10.1016/j.tcb.2022.07.005 35945077

[112] Kim, H. K. et al. The N-terminal methionine of cellular proteins as a degradation signal. *Cell* **156**, 158–169 (2014). 24361105 10.1016/j.cell.2013.11.031 PMC3988316

[113] Kim, J. M. N-terminal formylmethionine as a novel initiator and N-degron of eukaryotic proteins. *BMB Rep* **52**, 163–164 (2019). 30885288 10.5483/BMBRep.2019.52.3.069 PMC6476482

[114] Morrison, E. J. & Rissland, O. S. The generation and consequences of N-terminal proteoform diversity. *Cell Rep* **44**, 116275 (2025). 40946309 10.1016/j.celrep.2025.116275 PMC13082882

[115] Kremer, N. et al. CUL4A-DDB1-DCAF10 is an N-recognin for N-terminally acetylated Src kinases. *Nat Commun* **17**, 132 (2026). 41484149 10.1038/s41467-025-68074-9 PMC12775124

[116] Giglione, C., Fieulaine, S. & Meinnel, T. N-terminal protein modifications: bringing back into play the ribosome. *Biochimie* **114**, 134–146 (2015). 25450248 10.1016/j.biochi.2014.11.008

[117] Solbiati, J., Chapman-Smith, A., Miller, J. L., Miller, C. G. & Cronan, J. E. Jr Processing of the N termini of nascent polypeptide chains requires deformylation prior to methionine removal. *J Mol Biol* **290**, 607–614 (1999). 10395817 10.1006/jmbi.1999.2913

[118] Margolis, P. S. et al. Peptide deformylase in *Staphylococcus aureus*: resistance to inhibition is mediated by mutations in the formyltransferase gene. *Antimicrob Agents Chemother* **44**, 1825–1831 (2000). 10858337 10.1128/aac.44.7.1825-1831.2000 PMC89968

[119] Bandow, J. E. et al. The role of peptide deformylase in protein biosynthesis: a proteomic study. *Proteomics* **3**, 299–306 (2003). 12627383 10.1002/pmic.200390043

[120] Giglione, C., Vallon, O. & Meinnel, T. Control of protein life-span by N-terminal methionine excision. *EMBO J* **22**, 13–23 (2003). 12505980 10.1093/emboj/cdg007 PMC140049

[121] Bittner, L. M., Westphal, K. & Narberhaus, F. Conditional proteolysis of the membrane protein YfgM by the FtsH protease depends on a novel N-terminal degron. *J Biol Chem* **290**, 19367–19378 (2015). 26092727 10.1074/jbc.M115.648550 PMC4521054

[122] Wang, H. T. & Hur, S. Substrate recognition by TRIM and TRIM-like proteins in innate immunity. *Semin Cell Dev Biol* **111**, 76–85 (2021). 33092958 10.1016/j.semcdb.2020.09.013 PMC7572318

[123] Hatakeyama, S. TRIM family proteins: roles in autophagy, immunity, and carcinogenesis. *Trends Biochem Sci* **42**, 297–311 (2017). 28118948 10.1016/j.tibs.2017.01.002

[124] Hewawasam, G. et al. Psh1 is an E3 ubiquitin ligase that targets the centromeric histone variant Cse4. *Mol Cell* **40**, 444–454 (2010). 21070970 10.1016/j.molcel.2010.10.014 PMC2998187

[125] Sun, J. et al. TRIM52 positively mediates NF-kappaB to promote the growth of human benign prostatic hyperplasia cells through affecting TRAF2 ubiquitination. *Life Sci* **259**, 118380 (2020). 32898524 10.1016/j.lfs.2020.118380

[126] Pan, S. et al. TRIM52 promotes colorectal cancer cell proliferation through the STAT3 signaling. *Cancer Cell Int* **19**, 57 (2019). 30918473 10.1186/s12935-019-0775-4 PMC6419475

[127] Mu, X., Li, H., Zhou, L. & Xu, W. TRIM52 regulates the proliferation and invasiveness of lung cancer cells via the Wnt/beta‑catenin pathway. *Oncol Rep* **41**, 3325–3334 (2019). 31002351 10.3892/or.2019.7110

[128] Zhang, Y. et al. TRIM52 up-regulation in hepatocellular carcinoma cells promotes proliferation, migration and invasion through the ubiquitination of PPM1A. *J Exp Clin Cancer Res* **37**, 116 (2018). 29898761 10.1186/s13046-018-0780-9 PMC6001170

[129] Yang, W. et al. TRIM52 plays an oncogenic role in ovarian cancer associated with NF-kB pathway. *Cell Death Dis* **9**, 908 (2018). 30185771 10.1038/s41419-018-0881-6 PMC6125490

[130] Fan, W. et al. TRIM52: a nuclear TRIM protein that positively regulates the nuclear factor-kappa B signaling pathway. *Mol Immunol* **82**, 114–122 (2017). 28073078 10.1016/j.molimm.2017.01.003

[131] Fan, W. et al. TRIM52 inhibits Japanese Encephalitis Virus replication by degrading the viral NS2A. *Sci Rep* **6**, 33698 (2016). 27667714 10.1038/srep33698 PMC5035999

[132] Wang, K., Yang, Z., Wu, C. X. & Cao, J. Identification of TRIM52 as a potential biomarker in mortality risk assessment in patients with sepsis. *Hum Immunol* **85**, 111174 (2024). 39520802 10.1016/j.humimm.2024.111174

[133] Shulkina, A. et al. TRIM52 maintains cellular fitness and is under tight proteolytic control by multiple giant E3 ligases. *Nat Commun* **16**, 3894 (2025). 40274822 10.1038/s41467-025-59129-y PMC12022042

[134] Marasco, W. A. et al. Purification and identification of formyl-methionyl-leucyl-phenylalanine as the major peptide neutrophil chemotactic factor produced by *Escherichia coli*. *J Biol Chem* **259**, 5430–5439 (1984). 6371005

[135] Boulay, F., Tardif, M., Brouchon, L. & Vignais, P. The human *N*-formylpeptide receptor. Characterization of two cDNA isolates and evidence for a new subfamily of G-protein-coupled receptors. *Biochemistry* **29**, 11123–11133 (1990). 2176894 10.1021/bi00502a016

[136] Zhang, Q. et al. Circulating mitochondrial DAMPs cause inflammatory responses to injury. *Nature* **464**, 104–107 (2010). 20203610 10.1038/nature08780 PMC2843437

[137] Verhaar, B. J. H. et al. Plasma metabolite *N*-formylmethionine is associated with higher blood pressure in the multiethnic HELIUS Cohort and triggers vascular dysfunction. *Hypertension* **82**, 1916–1929 (2025). 10.1161/HYPERTENSIONAHA.125.24959 40959884

[138] Rogers, R. S. et al. Circulating *N*-lactoyl-amino acids and *N*-formyl-methionine reflect mitochondrial dysfunction and predict mortality in septic shock. *Metabolomics* **20**, 36 (2024). 38446263 10.1007/s11306-024-02089-z PMC10917846

[139] Kuley, R. et al. Mitochondrial *N*-formyl methionine peptides contribute to exaggerated neutrophil activation in patients with COVID-19. *Virulence* **14**, 2218077 (2023). 37248708 10.1080/21505594.2023.2218077 PMC10231045

[140] Sigurdsson, M. I. et al. Circulating *N*-formylmethionine and metabolic shift in critical illness: a multicohort metabolomics study. *Crit Care* **26**, 321 (2022). 36261854 10.1186/s13054-022-04174-y PMC9580206

[141] Kwon, W. Y. et al. Circulating mitochondrial N-formyl peptides contribute to secondary nosocomial infection in patients with septic shock. *Proc Natl Acad Sci USA* **118***.* e2018538118 (2021). 10.1073/pnas.2018538118 PMC8092466 33888581

[142] Kuley, R. et al. N-formyl methionine peptide-mediated neutrophil activation in systemic sclerosis. *Front Immunol* **12**, 785275 (2021). 35069556 10.3389/fimmu.2021.785275 PMC8766990

[143] Duvvuri, B. et al. Mitochondrial *N*-formyl methionine peptides associate with disease activity as well as contribute to neutrophil activation in patients with rheumatoid arthritis. *J Autoimmun* **119**, 102630 (2021). 33713887 10.1016/j.jaut.2021.102630 PMC8062236

[144] Cai, N. et al. Mitochondrial DNA variants modulate *N*-formylmethionine, proteostasis and risk of late-onset human diseases. *Nat Med* **27**, 1564–1575 (2021). 34426706 10.1038/s41591-021-01441-3

[145] Kwon, W. Y. et al. Removal of circulating mitochondrial *N*-formyl peptides via immobilized antibody therapy restores sepsis-induced neutrophil dysfunction. *J Leukoc Biol* **116**, 1169–1183 (2024). 39107254 10.1093/jleuko/qiae169

[146] Xu, T., Wang, Q., Wang, Q. & Sun, L. Mass spectrometry-intensive top-down proteomics: an update on technology advancements and biomedical applications. *Anal Methods* **16**, 4664–4682 (2024). 38973469 10.1039/d4ay00651h PMC11257149

[147] Lee, S. et al. tipNrich: a tip-based N-terminal proteome enrichment method. *Anal Chem* **93**, 14088–14098 (2021). 34615347 10.1021/acs.analchem.1c01722

[148] Ju, S. et al. iNrich, rapid and robust method to enrich N-terminal proteome in a highly multiplexed platform. *Anal Chem* **92**, 6462–6469 (2020). 32267142 10.1021/acs.analchem.9b05653

[149] Tanaka, F., Jones, T., Kubitz, D. & Lerner, R. A. Anti-formyl peptide antibodies. *Bioorg Med Chem Lett* **17**, 1943–1945 (2007). 17293112 10.1016/j.bmcl.2007.01.082

[150] Yang, J., Kim, S. Y. & Hwang, C. S. Delineation of the substrate recognition domain of MARCHF6 E3 ubiquitin ligase in the Ac/N-degron pathway and its regulatory role in ferroptosis. *J Biol Chem* **300**. 107731 (2024). 10.1016/j.jbc.2024.107731 PMC11460463 39216628

[151] Alagar Boopathy, L. R., Beadle, E., Garcia-Bueno Rico, A. & Vera, M. Proteostasis regulation through ribosome quality control and no-go-decay. *Wiley Interdiscip Rev RNA* **14**. e1809 (2023). 10.1002/wrna.1809 37488089

[152] Yang, J., Lee, Y. & Hwang, C. S. The ubiquitin–proteasome system links NADPH metabolism to ferroptosis. *Trends Cell Biol* **33**, 1088–1103 (2023). 37558595 10.1016/j.tcb.2023.07.003

